# Advances in the application of low-intensity pulsed ultrasound to mesenchymal stem cells

**DOI:** 10.1186/s13287-022-02887-z

**Published:** 2022-05-26

**Authors:** Peng Xia, Yi Shi, Xiaoju Wang, Xueping Li

**Affiliations:** Department of Rehabilitation Medicine, Nanjing First Hospital, Nanjing Medical University, Nanjing, 210006 China

**Keywords:** Mesenchymal stem cells, Low-intensity pulsed ultrasound, Microbubble, Cell transplantation

## Abstract

Mesenchymal stem cells (MSCs) are stem cells that exhibit self-renewal capacity and multi-directional differentiation potential. They can be extracted from the bone marrow and umbilical cord, as well as adipose, amnion, and other tissues. They are widely used in tissue engineering and are currently considered an important source of cells in the field of regenerative medicine. Since certain limitations, such as an insufficient cell source, mature differentiation, and low transplantation efficiency, are still associated with MSCs, researchers have currently focused on improving the efficacy of MSCs. Low-intensity pulsed ultrasound (LIPUS) has mechanical, cavitation, and thermal effects that can produce different biological effects on organs, tissues, and cells. It can be used for fracture treatment, cartilage repair, and stem cell applications. An in-depth study of the role and mechanism of action of LIPUS in MSC treatment would promote our understanding of LIPUS and promote research in this field. In this article, we have reviewed the progress in research on the use of LIPUS with various MSCs and comprehensively discussed the progress in the use of LIPUS for promoting the proliferation, differentiation, and migration of MSCs, as well as its future prospects.

## Introduction

Mesenchymal stem cells (MSCs) are stem cells that can be isolated from various tissues such as the bone marrow, adipose tissue, dental tissue, amnion, placenta, umbilical cord, and cord blood and have the potential for self-renewal and multi-directional differentiation [1]. Under certain conditions, MSCs can differentiate into a variety of organs or tissues, such as bone, fat, muscle, neurons, cardiomyocytes, and liver cells [2]. At present, MSCs have been widely used in tissue engineering and are currently considered important therapeutic agents in the field of regenerative medicine; their application is a current research hotspot [3]. However, the therapeutic effect of the transplantation of MSCs is limited because of their reduced cell activity, loss, and proliferation in tissues.

Low-intensity pulsed ultrasound (LIPUS) is a special type of ultrasound, whose output is generated in a low-intensity, pulsed wave mode. It can produce mechanical, cavitation, and thermal effects and is a noninvasive, safe, and simple physical therapy. It has been widely used for the treatment of fractures, arthritis, tendon and ligament injuries, and other diseases [4–6]. In recent years, researchers have found that LIPUS has an important effect on the biological activities of MSCs, such as proliferation, differentiation, and migration. LIPUS has gradually become an important technical approach used for enhancing the therapeutic effects of MSCs [7]. This article will review the application of LIPUS during the use of MSCs.

### Progress in research on MSCs

MSCs are pluripotent stem cells derived from the mesoderm that exhibit self-replication and multi-directional differentiation ability. MSCs can be obtained from a variety of sources and are easy to isolate, culture, and purify. After multiple passages in vitro, they still maintain the characteristics of stem cells and can differentiate under different induction conditions into cells of different tissue types, such as adipocytes, osteoblasts, chondrocytes, and muscle cells [8].

MSCs not only have the same strong self-renewal ability and multi-directional differentiation potential as other stem cells, but also have certain other characteristics. First, MSCs can play an immunomodulatory role through their interaction with immune cells and result in paracrine effects. In addition, MSCs have low immunogenicity; they do not strictly meet the compatibility requirements of allogeneic transplantation and do not easily undergo immune rejection. Furthermore, MSCs can home and adhere to inflammatory and tumor sites and thereby exhibit anti-inflammatory and anti-tumor effects [9–11]. Therefore, based on the above characteristics, MSCs can be used as ideal seed cells, for repairing the tissue and organ damage caused by aging and pathological changes, and could be broadly used in clinical applications for the treatment of autoimmune diseases and inflammation-related diseases.

Despite the success of MSC therapy in basic and clinical research, certain limitations, such as insufficient cell sources, mature differentiation, and low transplantation efficiency, are still associated with the use of MSCs. In addition, evidence shows that exogenous MSCs used for tissue regeneration result in a high level of apoptosis after transplantation [12]. Therefore, the long-term efficacy and safety of MSC therapy are still unclear. These issues are the main limiting factors for the use of MSCs in clinical practice. Therefore, the improvement in the efficacy of MSC therapy is the main limitation and focus of researchers studying stem cells.

### Application of LIPUS and the biological effect on MSCs

Ultrasound refers to a mechanical vibration wave with a frequency above 20 kHz that cannot cause a normal human auditory response. However, when it is propagated in a medium, it will cause changes in the rhythm and density of the medium. This change will produce changes in pressure levels, which will produce a good mechanical effect on cells. This effect on cells can increase the volume of cells and the permeability of cell membranes and promote the exchange of metabolites, thereby resulting in the regulation of the functions of cells; this can be used to treat trauma and diseases in nerves, muscles, and bones [13, 14].

LIPUS is an ultrasound wave with a frequency of 1–3 MHz and an intensity of < 1 W/cm^2^ that can provide low-intensity mechanical stimulation, produce micromechanical interactions with cells, induce intracellular biochemical effects, and ultimately lead to tissue repair and regeneration [15]. Studies have shown that LIPUS can effectively stimulate osteoblasts and promote bone formation and can be used for the treatment of fractures [16]. Previous studies have also demonstrated that the mechanical effects of LIPUS can activate stress receptor integrins and their mediated mechanochemical transduction pathways, inhibit the secretion of major cartilage degrading enzymes in osteoarthritis (OA), and inhibit cartilage vascular invasion and chondrocyte apoptosis. It promotes the synthesis of cartilage extracellular matrix and produces chondroprotective effects [17–19].

LIPUS is widely used clinically in musculoskeletal disorders. Based on evidences from some clinical studies, the US Food and Drug Administration(FDA) approved LIPUS as a treatment for fresh fractures and nonunions in 1994 and 2000, respectively [20–23]. In 2010, UK National Institute for Health and Care Excellence (NICE) issued a statement supporting the use of LIPUS to reduce fracture healing time and to provide clinical benefit, particularly in circumstances of delayed healing and nonunion [24]. In 2017, the clinical practice guidelines further summarized and standardized the application of LIPUS in fracture healing [4]. A recent review summarized the clinical application of LIPUS in fresh fractures, nonunions, and delayed fractures and believed that LIPUS is a safe and effective therapy to accelerate bone healing [25]. A randomized controlled trial using MRI to measure cartilage volume and thickness found that LIPUS increased medial tibial cartilage thickness in patients with mild and moderate OA, suggesting that LIPUS has a cartilage repair effect [26]. Another clinical study found that LIPUS significantly improved pain, function, and quality of life in patients with OA [27]. In addition, a review summarized the role of LIPUS in tendon–bone healing after anterior cruciate ligament (ACL) reconstruction in animal models, suggesting that LIPUS is a potential clinical therapeutic strategy to promote tendon–bone junction healing [28].

In recent years, an increasing number of studies have proven that LIPUS can affect the biological effects of MSCs, such as viability, proliferation, differentiation, and migration, through the mechanical stimulation of ultrasound waves [29–33]. Related studies have found that LIPUS can inhibit the apoptosis of 3D-cultured MSCs and enhance cell viability by affecting the expression of apoptosis-related genes [34]. In addition, LIPUS can promote the proliferation of MSCs by activating the extracellular regulated protein kinases 1/2(ERK1/2) and phosphatidylinositide 3-kinases (PI3K)/protein kinase B (Akt) signaling pathways [34]. Studies have shown that LIPUS can increase the levels of total collagen and glycosaminoglycan (GAG) in MSCs, improve the synthesis of the extracellular matrix, and promote the chondrogenic differentiation of MSCs [35]. Another study found that LIPUS can promote the migration of MSCs through the autophagy-regulated stromal cell gene-derived factor-1 (SDF-1) signaling pathway [36]. These studies, which showed that LIPUS could promote the biological functions of MSCs, will be discussed more thoroughly below.

### Application of LIPUS to various MSCs

**Fig. 1:**
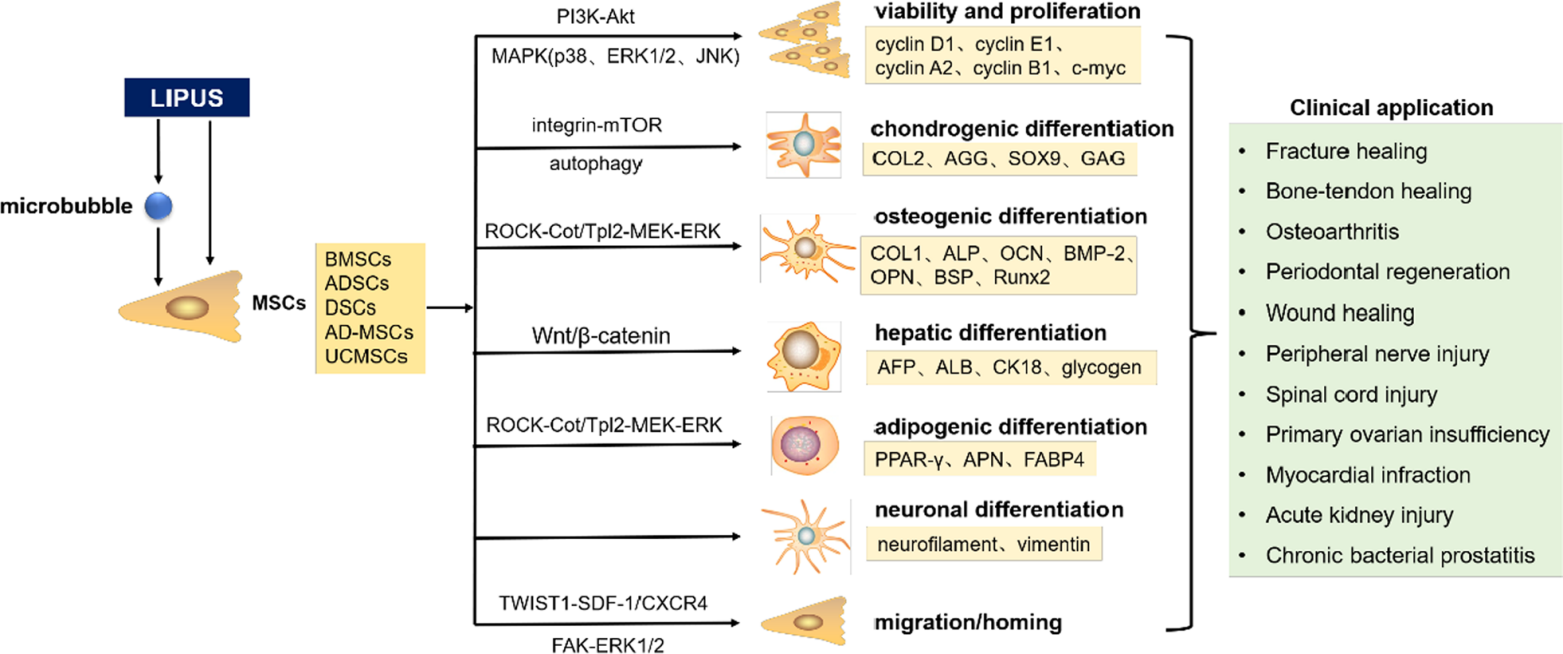
Application of LIPUS to various types of MSCs

LIPUS can act on a variety of MSCs, including bone marrow MSCs (BMSCs), adipose-derived stem cells (ADSCs), amnion-derived MSCs (AD-MSCs), and dental stem cells (DSCs). Based on the effects of these stem cells, LIPUS has become an important auxiliary tool for MSC therapy. The application of LIPUS in MSCs from these different sources is described in detail in Fig. 1 and Table 1.

**Table 1:** Application of LIPUS with or without microbubbles on various MSCs

Cell source	Study	LIPUS parameter			Effects on MSCs	Therapeutic application
		Intensity (mW/cm^2^)	Frequency (MHz)	Time (min)		
BMSCs	Yang et al.	Unclear	1.02	7.3	Viability	–
	Xie et al.	50/60	1.5	5/10	Proliferation	–
	Aliabouzar et al.	100	1.5	3	Chondrogenic differentiation	–
	Zhi et al.	100/150/200	1	15	Chondrogenic differentiation	–
	Xia et al.	40	3	20	Chondrogenic differentiation	–
	Wang et al.	50	3	20	Chondrogenic differentiation	–
	An et al.	100	1	10	Osteogenic differentiation	–
	Kusuyama et al.	30	1.5	20	Chondrogenic and osteogenic differentiation	–
	Li et al.	500/1000/1500	1	0.5/1	Hepatic differentiation	–
	Wei et al.	30	1.5	20	Migration	Fracture healing
	Chen et al.	30	0.25	20	Migration	Fracture healing
	Ning et al.	50	1	3	Viability and migration	Spinal cord injury
	Wang et al.	30	1.5	20	Migration and osteogenesis	Periodontal regeneration
	Xia et al.	50	3	20	Migration and chondrogenesis	Osteoarthritis
ADSCs	Wang et al.	70	0.5	1	Viability and proliferation	–
	Huang et al.	30	1.5	5	Proliferation	–
	Jiang et al.	100	1	8	Osteogenic differentiation	–
	Yue et al.	100	0.001	10	Osteogenic differentiation	–
	Fu et al.	30	1	20	Adipogenic differentiation	–
	Kang et al.	15	1	5	Promote angiogenesis of endothelial cells	Wound and tissue healing
	Yue et al.	20	1	10	Promote myelination of Schwann cells	Peripheral nerve injury
	Chen et al.	30 ± 5	1.5	20	Chondrogenesis and osteogenesis	Bone–tendon healing
PDLSCs	El-Bialy et al.	30	1.5	10	Proliferation and osteogenic differentiation	–
	Hu et al.	90	1.5	20	Osteogenic differentiation	–
	Kusuyama et al.	Unclear	unclear	20	Osteogenic differentiation	–
	Wang et al.	90	1.5	30	Migration	–
	Gao et al.	250	1	5	Proliferation	–
DPSCs	Gao et al.	750	1	5	Proliferation	–
ABMSCs	Lim et al.	50	1	10	Viability and osteogenic differentiation	–
GMSCs	El-Bialy et al.	30	1.5	10	Neuronal differentiation	–
AD-MSCs	Ling et al.	30	0.25	30	Proliferation	–
	Ling et al.	30	0.25	30	Promote ovarian regeneration	Primary ovarian insufficiency
UCMSCs	Yoon et al.	25–35	0.04	1.67/5/10	Viability and proliferation	–
	Chen et al.	30/50	1.5	5	Proliferation	–

LIPUS combined with microbubbles						
BMSCs	Aliabouzar et al.	30	1.5	1/3/5	Proliferation and chondrogenic differentiation	–
	Osborn et al.	30	1.5	3	Proliferation and osteogeneic Differentiation	–
	Li et al.	600	1	0.5	Migration	Myocardial infraction
	Wang et al.	600	1	0.5	Migration	Acute kidney injury
	Yi et al.	23	1	5	Migration	Chronic bacterial prostatitis

LIPUS: low-intensity pulsed ultrasound; MSCs: mesenchymal stem cells; BMSCs: bone marrow MSCs; ADSCs: adipose-derived stem cells; AD-MSCs: amnion-derived MSCs; PDLSCs: periodontal ligament stem cells; DPSCs: dental pulp stem cells; ABMSCs: alveolar bone mesenchymal stem cells; GMSCs: gingival mesenchymal stem cells; UCMSCs: umbilical cord MSCs

#### Application of LIPUS to BMSCs

BMSCs are the earliest discovered MSCs. MSCs isolated from the bone marrow have advantages such as ease of proliferation and high differentiation potential and are currently the most studied MSCs that are regarded as the standard cells used during the clinical application of MSCs [37]. LIPUS has been shown to increase the viability of BMSCs and promote proliferation. Yang et al. used LIPUS to stimulate rat BMSCs and found that LIPUS increased the survival rate of BMSCs by 19.57%. After optimizing the LIPUS parameters using a genetic algorithm-based back-propagation neural network, the optimal conditions were 6.92 V, 1.02 MHz, and 7.3 min, and the viability of BMSCs was further improved by about 5.36% [38]. Xie et al. used LIPUS to stimulate human BMSCs. They found that LIPUS at an intensity of 50 or 60 mW/cm^2^ could drive cells from the G0/G1 phase to the S phase and G2/M phase and significantly promote the proliferation of BMSCs after a stimulation period of 5 or 10 min. At the same time, LIPUS could increase the phosphorylation of PI3K/Akt and significantly up-regulate the expression of cyclin D1, while the PI3K inhibitor blocked the above effects and reduced the LIPUS-mediated proliferation of BMSCs. It was suggested that LIPUS might promote the proliferation of BMSCs by activating the PI3K/Akt signaling pathway and up-regulating the expression of cyclin D1 [29].

The effect of LIPUS on the differentiation of BMSCs has also been confirmed in many studies. Zhi et al. and Aliabouzar et al. found that LIPUS could promote the proliferation of BMSCs and increase the content of GAG, type II collagen (COL2), and total collagen. LIPUS reportedly has a certain effect on the chondrogenic differentiation of BMSCs [39, 40]. In our previous study, we added transforming growth factor-β (TGF-β) to the culture medium of BMSCs and then provided LIPUS stimulation at different intensities (20, 30, 40, and 50 mW/cm^2^). We found that LIPUS could increase the expression of COL2, aggrecan, and sex-determining region of Y chromosome-related high-mobility-group box gene 9 (SOX9) in BMSCs, while reducing the expression of type I collagen (COL1). The above effects of LIPUS were blocked after the administration of integrin and mammalian target of rapamycin (mTOR) inhibitors. A further in-depth study also found that autophagy was involved in the regulation of chondrogenic differentiation of BMSCs, and LIPUS could inhibit the autophagy of BMSCs after chondrogenic induction, while increasing the synthesis of the extracellular matrix. These results indicate that LIPUS can promote the chondrogenic differentiation of BMSCs through the regulation of the integrin-mTOR signaling pathway and autophagy [40–42]. An et al. used stimulated rat BMSCs with LIPUS at an intensity of 100mW/cm^2^ and found that LIPUS could increase the expression of osteogenesis-related genes such as COL1, alkaline phosphatase (ALP), osteocalcin (OCN), bone morphogenetic protein-2 (BMP-2), and osteopontin (OPN) and promote the osteogenic differentiation of BMSCs [43]. The study by Kusuyama et al. also found that LIPUS at an intensity of 30 mW/cm^2^ could reduce the genetic expression of peroxisome proliferators-activated receptors-γ2 (PPAR-γ2) and fatty acid-binding protein 4 (FABP4), inhibit adipogenic differentiation, and simultaneously increase cell calcification and the expression of Runx2 and OCN, thereby promoting osteogenic differentiation. The above effects can be blocked by specific inhibitors of mitogen-activated protein kinase kinase 1 (MAPKK1 or MEK1) and Cot/Tpl2, key regulators of ERK phosphorylation. It has been shown that LIPUS can inhibit the adipogenic differentiation of BMSCs and promote osteogenic differentiation through the Rho-associated kinase (ROCK)-Cot/Tpl2-MEK-ERK signaling pathway [44]. Li et al. treated human BMSCs with hepatocyte growth factor (HGF) and then applied LIPUS at an intensity of 0.5–1.5 W/cm^2^. They found that LIPUS could significantly increase the levels of liver markers α-fetoprotein (AFP), cytokeratin 18 (CK18), albumin (ALB), and glycogen and increase the expression levels of Wnt1, β-catenin, c-myc, and cyclin D1 in BMSCs, while Wnt inhibitors could attenuate these effects. It was proven that LIPUS could promote the differentiation of HGF-induced BMSCs into hepatocytes through the Wnt/β-catenin signaling pathway [45].

The role of LIPUS in promoting the migration and homing of BMSCs in order to repair damaged tissue has also been confirmed. Wei et al. applied LIPUS at an intensity of 30 mW/cm^2^ to rat BMSCs in vitro and in vivo. They found that LIPUS could increase the expression levels of SDF-1 and CXC chemokine receptor type 4 (CXCR4) in BMSCs and promote the migration of BMSCs to the fracture site. In addition, the results showed that the SDF-1 inhibitor could inhibit the migration of BMSCs and attenuate the positive effect of LIPUS on the repair of fractured bones by BMSCs [32]. Chen et al. used LIPUS at a strength of 30 mW/cm^2^ to stimulate BMSCs in vitro. In in vivo experiments, BMSCs were injected into rats with femoral defects, followed by a LIPUS intervention. The results showed that LIPUS could promote the migration of BMSCs and improve the fracture healing rate, while the intervention with focal adhesion kinase (FAK) and ERK1/2 inhibitors reduced the LIPUS-induced migration of BMSCs. It was shown that LIPUS could promote the migration of BMSCs in vitro and in vivo, and one of the mechanisms for this process could be related to the activation of the FAK-ERK1/2 signaling pathway [46]. Rat BMSCs were subjected to LIPUS at different intensities (10, 30, 50, 70 mW/cm^2^) in vitro by Ning et al. They found that LIPUS could improve the cell viability, migration rate, and expression levels of neurotrophic factors in BMSCs. Then, BMSCs were transplanted into the epicenter of the injured spinal cord in rats and subjected to a LIPUS intervention at an intensity of 50 mW/cm^2^. It was found that in comparison with the BMSC transplantation group alone, the formation of the spinal cord cavity was reduced in the LIPUS combined with BMSC transplantation group. In addition, the levels of brain-derived neurotrophic factor (BDNF) and nerve growth factor (NGF) were higher in the epicentral region, while the expression of neurotrophic receptors was also enhanced, and the motor function of rats was significantly improved. It has been shown that the combination of transplantation of BMSCs and LIPUS could promote the functional recovery of spinal cord injury more effectively [47]. The latest study by Wang et al. explored the effect of LIPUS on the homing of BMSCs and its potential to promote alveolar bone regeneration. They found that a LIPUS intervention at an intensity of 30 mW/cm^2^ for 3 or 7 days could significantly promote the migration and homing of BMSCs to the alveolar bone defect zone, increase the expression of COL1 and OPN, and promote bone formation. These results demonstrated that LIPUS could enhance BMSC-based periodontal alveolar bone regeneration [48]. In our previous study, we stimulated rat BMSCs with LIPUS in vitro and found that LIPUS at an intensity of 50 mW/cm^2^ could significantly activate autophagy, increase the expression of SDF-1 and CXCR4, and promote the migration of BMSCs, while an autophagy inhibitor could attenuate these effects of LIPUS. In an in vivo study, we injected BMSCs into the joint cavity and then subjected the cells to a LIPUS intervention. The results showed that in comparison with BMSCs alone, the combination of LIPUS and BMSCs could significantly promote OA cartilage repair, while an autophagy inhibitor could attenuate the positive effects of LIPUS on BMSCs during OA cartilage repair. It was shown that LIPUS could promote BMSC migration and repair OA cartilage via the regulation of autophagy [49].

The studies discussed above have shown that LIPUS can increase the activity of BMSCs and promote proliferation, which may be related to the activation of the PI3K/Akt signaling pathway and the up-regulation of cell proliferation-related genes. At the same time, LIPUS can inhibit the adipogenic differentiation of BMSCs and promote osteogenic, chondrogenic, and hepatocyte differentiation, which mainly involves the down-regulation of adipogenic genes, up-regulation of osteogenic and chondrogenic genes, regulation of autophagy, activation of the ROCK-Cot/Tpl2-MEK-ERK and Wnt/β-catenin signaling pathways, and other related mechanisms. In vivo studies also showed that LIPUS can promote the therapeutic effects of BMSCs during fracture healing, alveolar bone regeneration, OA cartilage repair, and functional recovery from spinal cord injury. These processes are related to the regulation of autophagy, activation of the SDF-1/CXCR4 and FAK-ERK1/2 signaling pathways, and the up-regulation of neurotrophic factors. In the future, clinical trials need to be conducted to verify the therapeutic effect of the combination of LIPUS and BMSCs in the above diseases.

#### Application of LIPUS to ADSCs

The use of BMSCs is limited by the invasiveness of the isolation process and the low percentage of BMSCs in the bone marrow. ADSCs are MSCs that are easier to obtain than BMSCs; 300 times the number of BMSCs can be obtained with a minimally invasive method. Other adipose tissues discarded after surgery also represent an important source, and the method for their isolation is simpler than that for BMSCs, and no ethical issues are associated with it [50].

The effects of LIPUS on the viability, proliferation, and differentiation of ADSCs have been confirmed by many studies. Wang et al. used LIPUS at an intensity of 70 and 210 mW/cm^2^ to stimulate human ADSCs. They found that a high dose of LIPUS (210 m W/cm^2^) promoted the apoptosis of ADSCs and significantly increased the phosphorylation of p38 MAPK, and a low dose (70 mW/cm^2^) of LIPUS increased the viability of ADSCs and significantly decreased the phosphorylation of p38 MAPK, while the p38 MAPK inhibitor could reverse the apoptotic effect induced by a high-dose LIPUS. The results showed that LIPUS had dose-dependent effects on ADSCs and suggested that p38 MAPK plays a key role in mediating the effect of LIPUS on ADSCs [51]. Huang et al. also used LIPUS at an intensity of 30 mW/cm^2^ to stimulate human ADSCs and found that the number of ADSCs increased after LIPUS intervention, but there was no significant change in the levels of cell surface markers. At the same time, LIPUS prolonged the G1 and S phases of the cell cycle, increased the expression of cell proliferation-related genes (CCND1 and c-myc), SDF-1, and other cytokine genes, and decreased the apoptosis rate. These results indicated that LIPUS could promote the proliferation of human ADSCs and facilitate the maintenance of their stem cell properties [52]. Jiang et al. and Yue et al. used LIPUS at an intensity of 100 mW/cm^2^ to stimulate rat or mouse ADSCs and found that LIPUS could lead to the formation of mineralized nodules in ADSCs in vitro and increase the expression of osteogenesis-related genes such as Runx2, OCN, ALP, OPN, and bone sialoprotein (BSP). These results suggested that LIPUS could induce the osteogenic differentiation of ADSCs in vitro [53, 54]. Fu et al. stimulated mouse ADSCs with LIPUS at an intensity of 30 mW/cm^2^ after adipogenic induction. The results showed that LIPUS could up-regulate the levels of the adipogenic factor PPAR-γ and adiponectin (APN) and promote the adipogenic differentiation of ADSCs [55].

Angiogenesis is crucial and beneficial for wound healing and tissue regeneration. Kang et al. prepared scaffolds composed of collagen and hyaluronic acid and then co-cultured human ADSCs and human umbilical vein endothelial cells in the scaffolds following LIPUS stimulation at an average intensity of 15 mW/cm^2^. The results showed that LIPUS significantly enhanced cell growth on scaffolds and increased the mRNA expression of CD31 and vascular endothelial cadherin (VE-cadherin). This showed that LIPUS could promote the angiogenesis ability and therapeutic potential of the scaffold-based co-culture of ADSCs/human umbilical vein endothelial cells (HUVECs) [56].

Peripheral nerve injury is a common disease, and current research is focused on the promotion of myelination of Schwann cells, in order to enable the nerves to perform their functions efficiently. Yue et al. co-cultured ADSCs with Schwann cells and then administered LIPUS at an intensity of 20 mW/cm^2^. The results showed that the expression of epidermal growth factor receptor 3 (EGFR3)/ErbB3, neuregulin1 (NRG1), early growth response protein 2 (EGR2)/Krox20, and myelin basic protein (MBP) was significantly up-regulated and their up-regulation was more significant after LIPUS intervention. This showed that ADSCs could up-regulate the myelination markers of Schwann cells, and LIPUS can enhance this effect and show potential to promote nerve regeneration [57].

Another study explored the role of LIPUS in the promotion of bone–tendon healing by ADSCs. Chen et al. randomly divided adult rabbits with partial patella resection into the control group, LIPUS group, ADSC group, and LIPUS + ADSC group. In the LIPUS + ADSC group, autologous ADSCs were transplanted to the healing site and then stimulated by LIPUS at an average intensity of 30 ± 5 mW/cm^2^. The patella–patellar tendon junctions were collected at 8 and 16 weeks after the operation. The results showed that in comparison with other groups, the bone volume fraction, trabecular thickness, and the number of trabecular bones at the healing site were significantly increased. A greater extent of formation and maturity of the fibrocartilage layer and new bone were observed, and the failure load and stiffness were significantly increased in the LIPUS + ADSC group. These results suggested that the transplantation of autologous ADSCs with LIPUS intervention resulted in better bone–tendon healing quality [58].

Human ADSCs have been shown to treat OA in early preclinical and clinical studies. Nasb et al. are conducting a randomized controlled clinical study in which patients will be randomly assigned to receive ADSCs, LIPUS, or ADSCs + LIPUS. The blinded assessments were performed at 1 month, 3 months, and 6 months after the intervention. This study will be the first to investigate the safety and efficacy of LIPUS for the treatment of ADSCs in patients with OA. The results are expected to provide evidence for the effectiveness of LIPUS in improving treatment with ADSCs [59].

The above studies have shown that LIPUS can increase the activity of ADSCs and promote proliferation in a dose-dependent manner. These effects are related to the activation of the MAPK signaling pathway and the up-regulation of cell proliferation-related genes. In addition, LIPUS can promote the osteogenic and adipogenic differentiation of ADSCs by up-regulating osteogenic genes and adipogenic genes, respectively, and enhance the effects of ADSCs on the promotion of angiogenesis, nerve regeneration, and bone–tendon healing. Certain research teams have started clinical trials involving the combination of LIPUS and ADSCs for the treatment of OA, to determine the ability of LIPUS to promote the treatment of OA with ADSCs. This would provide important experimental evidence regarding the application of a combination of LIPUS and ADSCs in humans.

#### Application of LIPUS to DSCs

DSCs are MSCs extracted from certain tissues in the teeth, such as the craniofacial bone, dental pulp, periodontal ligament, dental follicle, tooth germ, apical papilla, oral mucosa, gingiva, and periosteum, and have self-renewal and multi-directional differentiation potential[60]. Eight kinds of DSCs have been isolated and identified to have been derived at different stages of tooth development, and these include the periodontal ligament stem cells (PDLSCs), dental pulp stem cells (DPSCs), gingival MSCs (GMSCs), stem cell populations from human exfoliated deciduous teeth (SHEDs), follicular cell progenitor cells (DFPCs), alveolar bone MSCs (ABMSCs), dental papilla stem cells (SCAPs), and tooth germ cells (TGPCs) [61].

Current studies related to LIPUS mainly focus on PDLSCs. El-Bialy et al. divided PDLSCs into three groups, including the control group, 5-min LIPUS intervention group, and 10-min LIPUS intervention group. They found that after subjecting DSCs to a 4-week LIPUS intervention for 10 min per day at an intensity of 30 mW/cm^2^, the expression levels of ALP, cyclin D1, and nucleostemin (NCT) were increased. The results indicated that LIPUS might enhance the pluripotent properties of PDLSCs by up-regulating stemness marker NCT, which demonstrates the potential role of LIPUS in periodontal tissue regeneration [62]. Hu et al. isolated PDLSCs from adolescent premolars and then applied different intensities of LIPUS. They found that LIPUS at an intensity of 90 mW/cm^2^ significantly increased ALP activity, OCN production, and calcium nodule formation and up-regulated the mRNA expression levels of Runx2 and integrin β1, while these effects were decreased after the administration of the integrin β1 inhibitor. The results demonstrated that LIPUS could promote the osteogenic differentiation of PDLSCs by up-regulating integrin and Runx2 [63]. Kusuyama et al. extracted PDLSCs from three healthy third molars and then induced PDLSC differentiation using recombinant BMP9 and a LIPUS intervention. The results showed that LIPUS promoted the BMP9-induced osteogenic differentiation of PDLSCs and simultaneously blocked the inhibitory effects of porphyromonas gingivalis-derived LPS (LPS-PG) and interleukin-1β (IL-1β) on the osteogenic differentiation of PDLSCs. These results suggested that LIPUS is an effective tool that promotes the osteogenic differentiation of PDLSCs under inflammatory conditions [64]. Wang et al. also performed a LIPUS intervention on human PDLSCs, and the results showed that LIPUS at an intensity of 90 mW/cm^2^ significantly increased the expression of twist family bHLH transcription factor 1 (TWIST1) and SDF-1 and enhanced PDLSCs migration, while the knockdown of TWIST1 decreased SDF-1 expression and the cell migration of PDLSCs. It was suggested that the TWIST1-SDF-1/CXCR4 signaling pathway was involved in LIPUS-promoted PDLSC migration, which may be one of the mechanisms by which LIPUS-mediated periodontal tissue regeneration occurs [65].

The effect of LIPUS on the proliferation of DPSCs and its mechanism of action has been confirmed in some studies. Gao et al. compared the effects of LIPUS on proliferation and MAPK signaling in rat DPSCs versus PDLSCs. DPSCs or PDLSCs were stimulated with LIPUS at an intensity of 250 or 750 mW/cm^2^, and it was found that LIPUS at an intensity of 750 mW/cm^2^ could significantly promote the proliferation of DPSCs, and LIPUS at an intensity of 250 mW/cm^2^ could significantly promote the proliferation of PDLSCs. At the same time, LIPUS could activate ERK1/2 signaling in DPSCs and c-Jun N-terminal kinase (JNK) signaling in PDLSCs and simultaneously activate p38 signaling, while ERK1/2 inhibitors inhibited the LIPUS-mediated stimulation of DPSCs, and JNK and p38 inhibitors inhibited the LIPUS-stimulated proliferation of PDLSCs. It was shown that LIPUS promotes the proliferation of DPSCs and PDLSCs in an intensity and cell-specific manner by activating different MAPK pathways [66]. A further study by Gao et al. also found that Piezo membrane ion channels that were sensitive to mechanical stimulation existed in both DPSCs and PDLSCs. After 24 h of LIPUS intervention in both cells, the level of Piezo protein was significantly increased in DPSCs, but no obvious impact was observed in PDLSCs. The Piezo inhibitor could significantly inhibit the proliferation of LIPUS-stimulated DPSCs, but not PDLSCs. The Piezo inhibitor could also affect MAPK signaling in DPSCs and PDLSCs, and its effect on the phosphorylation level of ERK1/2 K is the most prominent. It was suggested that the stimulation of DPSC proliferation by LIPUS involves the Piezo-mediated regulation of ERK1/2 signaling [67].

The effect of LIPUS on ABMSCs and GMSCs has also been confirmed. Lim et al. used LIPUS at an intensity of 50mW/cm^2^ to intervene in human ABMSCs. They found that LIPUS could significantly increase the viability of ABMSCs, enhance the gene expression levels of CD29, CD44, ALP, COL1, and OCN, and increase the formation of mineralized nodules, indicating that LIPUS can enhance the cell viability and osteogenic differentiation of ABMSCs [33]. El-Bialy et al. isolated GMSCs from the proximal interdental papilla of human premolars, induced their differentiation into neural lineages with a neural induction medium, and then stimulated them with LIPUS at an intensity of 30 mW/cm^2^. The results showed that GMSCs could differentiate into neural lineages and that LIPUS could increase the mRNA expression of neural differentiation markers such as neurofilament and vimentin and reduce the mRNA expression of nucleostemin in GMSCs. It was demonstrated that LIPUS could promote the neural differentiation of GMSCs and has the potential to be an adjunct tool for the tissue engineering of the dental pulp and other craniofacial nerves [68].

The above studies indicated that LIPUS could have effects on different kinds of DSCs, such as PDLSCs, DPSCs, GMSCs, and ABMSCs. Among these, LIPUS can enhance the pluripotent properties of PDLSCs and promote the osteogenic differentiation and migration of PDLSCs; these effects are related to the up-regulation of stemness markers and osteogenic genes and the activation of the TWIST1-SDF-1/CXCR4 signaling pathway. In addition, LIPUS can promote the proliferation of DPSCs via a mechanism related to the Piezo-mediated activation of the MAPK signaling pathway. LIPUS can also improve the viability and osteogenic differentiation of ABMSCs and promote the neural differentiation of GMSCs; these effects are related to the up-regulation of osteogenic and neural differentiation genes. However, the effect of LIPUS on other types of DSCs such as DFPCs, SHEDs, SCAPs, and TGPCs has not been reported, and further studies are needed to confirm these findings.

#### Application of LIPUS to AD-MSCs

The amnion is derived from an early embryonic product that is considered medical waste and is easy to obtain. AD-MSCs are derived from the mesoderm and ectoderm during embryonic development and are distributed in the collagen matrix under the amniotic epithelial monolayer, with paracrine and autocrine properties. It can secrete a variety of biologically active substances [69]. Ling et al. isolated and cultured human AD-MSCs from the amnion of the term placenta and then stimulated the cells with LIPUS at an intensity of 30 mW/cm^2^. The results showed that LIPUS could promote the growth of cells from the G0/G1 phase to the S phase and G2/M phase and promote proliferation. At the same time, it can activate ERK1/2 and Akt and up-regulate the expression of cyclin D1, cyclin E1, cyclin A2, and cyclin B1, while the ERK1/2 inhibitor and PI3K inhibitor could significantly reduce the LIPUS-induced phosphorylation of ERK1/2 and Akt and inhibit the LIPUS-induced proliferation of AD-MSCs. It was shown that LIPUS can promote the proliferation of AD-MSCs, and the ERK1/2 and PI3K/Akt signaling pathways may play an important role in this process [35]. Ling et al. further explored the effect of LIPUS-pretreated human AD-MSC transplantation on chemotherapy-induced primary ovarian insufficiency (POI) in rats. The POI rat model was established via the intraperitoneal injection of cyclophosphamide. AD-MSCs were isolated from the human amnion and treated with LIPUS at an intensity of 30 mW/cm^2^ for 5 consecutive days and then injected into the tail vein of POI rats. The results showed that LIPUS could promote the secretion of growth factors in AD-MSCs, and the transplantation of AD-MSCs and LIPUS-pretreated AD-MSCs could increase the body weight and reproductive organ weight of POI rats, improve ovarian function, and reduce reproductive organ damage. The transplantation of AD-MSCs increased the Bcl-2/Bax ratio and reduced the extent of granulosa cell apoptosis and ovarian inflammation induced by ovarian chemotherapy, which was more pronounced after pre-treatment with LIPUS. It indicated that both AD-MSC transplantation and LIPUS-pretreated AD-MSC transplantation could repair ovarian damage and improve ovarian function in POI rats induced via chemotherapy, while the LIPUS pre-treatment of AD-MSC transplantation was more beneficial for reducing inflammation, improving the local microenvironment, and inhibiting the chemotherapy-induced apoptosis of granulosa cells in the ovarian tissue of POI rats [70].

The above studies showed that LIPUS could promote the proliferation of AD-MSCs, and this effect was related to the up-regulation of cyclins and the activation of the ERK1/2 and PI3K/Akt signaling pathways. In vivo experiments also showed that LIPUS could enhance the therapeutic effect of AD-MSCs in POI. At present, there are few related studies on the application of LIPUS to AD-MSCs. In the future, it is necessary to study further the role of LIPUS in AD-MSC differentiation, migration, and treatment of other diseases and to explore possible related mechanisms.

#### Application of LIPUS to UCMSCs

UCMSCs refer to pluripotent stem cells that exist in neonatal umbilical cord tissues. Human UCMSCs can be successfully expanded using the culture system for inactivated umbilical cord serum. The cultured cells have the basic characteristics of MSCs and can differentiate into a variety of tissue cells and have broad clinical application prospects [71]. It has been reported that UCMSCs isolated from the human umbilical cord exhibit an enhanced cell content, lower immunogenicity, and proliferation ability than BMSCs and have the advantages such as ease of sampling and lack of ethical controversies associated with their use. In addition, they maintain their high differentiation capacity at the early embryonic stage; this results in the more rapid delivery of a clinically effective cell dose of UCMSCs, as compared to the delivery of the same dose of BMSCs [72–74].

The effect of LIPUS on the proliferation and differentiation of UCMSCs has been confirmed in previous studies. Yoon et al. isolated UCMSCs from human umbilical cords and then administered a LIPUS intervention at an intensity of 25–35mW/cm^2^ to P0–P4 generation UCMSCs. The results showed that the administration of a LIPUS intervention for 3–5 days could significantly improve the overall proliferation rate of UCMSCs, which peaked in the P3 generation. In addition, a LIPUS intervention for 100 s had no significant effect on the cell viability of UCMSCs in the P0 generation, but could significantly promote proliferation, while a LIPUS intervention for 300 s and 600 s could significantly reduce cell viability, but had no significant effect on proliferation. It was suggested that LIPUS could promote proliferation without altering the cell viability of UCMSCs [75]. UCMSCs were subjected to LIPUS at different intensities (30, 50, 80 mW/cm^2^) by Chen et al. Each LIPUS intervention group was subjected to ultrasound treatment for 5, 10, and 20 min/day, for 5 consecutive days. The results showed that LIPUS could significantly improve the cell proliferation of UCMSCs, and a LIPUS intervention at an intensity of 50 mW/cm^2^ for 5 min/day provided the most notable results. However, the cell proliferation of UCMSCs was inhibited with an increase in the intervention intensity and time. In addition, a LIPUS intervention at an intensity of 30 mW/cm^2^ for 5 min/day could significantly increase the expression levels of COL2 and GAG in UCMSCs cultured with a chondrogenic medium and promote the chondrogenic differentiation of UCMSCs, while LIPUS interventions at other intensities and periods had no significant effect on the chondrogenic differentiation of USMSCs. It was found that a LIPUS intervention with appropriate parameters could promote the proliferation of UCMSCs, but the proliferative capacity of cells decreased with an increase in the intervention intensity and time. In addition, under the synergistic effect of chondro-inducing factors, the administration of LIPUS with appropriate parameters could promote the chondrogenic differentiation of UCMSCs [76].

The above studies have shown that the administration of LIPUS at an appropriate intensity for a certain duration can promote the proliferation and chondrogenic differentiation of UCMSCs, but the mechanism needs to be further elucidated. In addition, the effect of LIPUS on the migration and other differentiation abilities of UCMSCs (such as osteogenic differentiation and adipogenic differentiation) is still unknown. There is no relevant experimental report on the application of a combination of LIPUS and UCMSCs for disease treatment, and further research needs to be conducted in the future.

### Application of a combination of LIPUS and microbubbles in MSCs

**Fig. 2:**
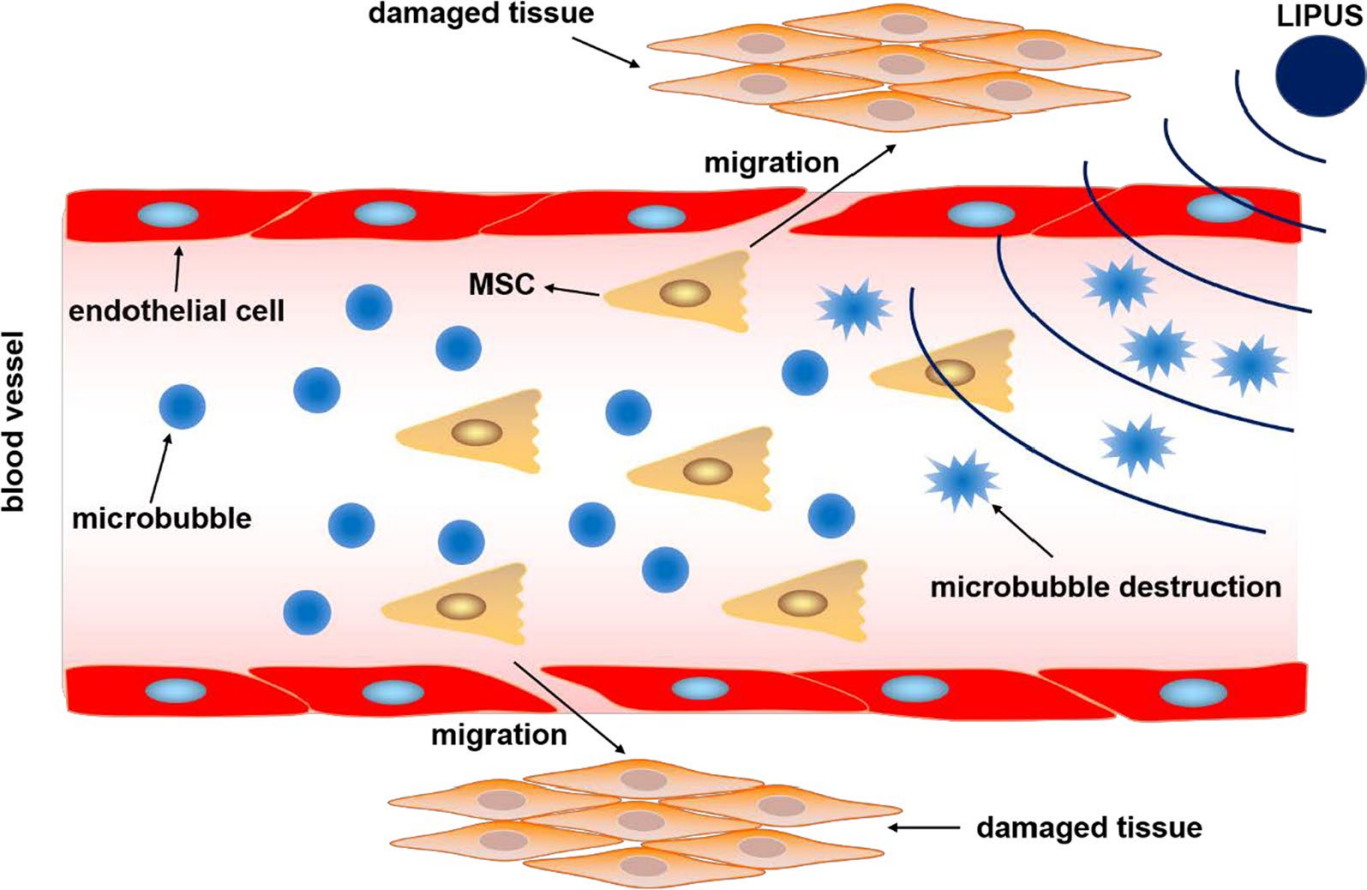
Application of a combined LIPUS and microbubbles to MSCs

Ultrasound-targeted microbubble destruction (UTMD) is a new technology in the field of ultrasonic therapy and a current research hotspot. A microbubble is an ultrasound imaging contrast agent that is injected either locally or through a peripheral vein; subsequently, ultrasound is used to rupture the microbubble to produce a cavitation effect when it flows through the target site, thereby producing a targeted therapeutic effect [77] (Fig. 2). An increasing number of studies have confirmed that UTMD can improve the efficiency of MSC transplantation and provide a new solution to solve problems related to survival rate, differentiation rate, and targeting ability in MSC transplantation, thereby showing its broad application prospects [78–83] (Table 1).

The role of the combination of LIPUS and microbubbles in the proliferation and differentiation of MSCs has been confirmed by studies. Aliabouzar et al. administered LIPUS alone or in combination with a microbubble into human BMSCs cultured in 3D-printed scaffolds and found that the combination of LIPUS and the microbubble intervention at an intensity of 30mW/cm^2^ increased the cell number of MSCs by 40%, compared with the increase in cell number by 18% that was observed using LIPUS alone. In addition, the combination of LIPUS with the microbubble could increase the GAG content in BMSCs by 17% (5% when treated with LIPUS alone) and the COL2 content by 78% (44% when treated with LIPUS alone). It was shown that the combination of LIPUS and microbubbles could significantly promote the proliferation and chondrogenic differentiation of BMSCs, compared to that observed with LIPUS alone [84]. The study by Osborn et al. also found that the combination of LIPUS at an intensity of 30 mW/cm^2^, 3 min/day with the microbubble intervention for 1, 3, and 5 days significantly promoted the proliferation of human BMSCs cultured in 3D-printed scaffolds, and LIPUS intervention alone or the combined LIPUS and microbubble intervention could increase the total protein content, calcium deposition, and ALP activity in human BMSCs. It was shown that the combination of LIPUS and microbubbles could promote the proliferation and osteogenic differentiation of BMSCs [85].

The application of the combination of LIPUS and microbubbles has also been confirmed to be effective for the homing of MSCs for the repair of damaged tissues. Li et al. used a combined LIPUS (intensity 0.6 W/cm^2^) and microbubble intervention in vitro for human and rat BMSCs and simultaneously administered myocardial infarction rats a BMSC injection and a combined LIPUS and microbubble intervention in vivo. The in vitro results showed that the combination of LIPUS with microbubbles could significantly increase the expression of SDF-1 and CXCR4 in BMSCs, and the in vivo results showed that the combination of LIPUS with microbubbles could increase the number of homing BMSCs in the ischemic myocardium and increase the expression levels of SDF-1 and CXCR4. It was shown that the combination of LIPUS and microbubbles could promote the homing of BMSCs to repair the ischemic myocardium through the SDF-1/CXCR4 signaling pathway [86]. Wang et al. cultured rat BMSCs in vitro, performed CXCR4 transfection modifications, and intervened with a combination of LIPUS (intensity 0.6 W/cm^2^) and a microbubble. They found that the combination of LIPUS and the microbubble could improve the transfection efficiency of CXCR4. CXCR4-transfected modified BMSCs were injected into a rat model of acute kidney injury (AKI) through the tail vein, and the results showed that the migration ability of the modified BMSCs was significantly increased after CXCR4 transfection, and the number of BMSCs homing into the rat AKI kidney tissue was found to be significantly increased. This effect was more notable in BMSCs treated with a combination of LIPUS and microbubbles. It was suggested that the combination of LIPUS and microbubbles could promote the homing of BMSCs to AKI tissues via elevating the expression of CXCR4 [87]. Yi et al. established a rat model of chronic bacterial prostatitis (CBP) and then administered BMSCs, BMSCs + LIPUS (intensity 23mW/cm^2^), and BMSCs + LIPUS + microbubble treatment. They found that the number of BMSCs found in the prostate gland of the BMSCs + LIPUS + microbubble group was more than that of the CBP control group, the BMSC group, and the BMSCs + LIPUS group. In addition, the levels of inflammatory cell infiltration, fibrosis, tumorlike epithelial proliferation, and IL-1β and tumor necrosis factor-α (TNF-α) expression were significantly decreased in the BMSCs + LIPUS + microbubble group. It was shown that the homing of BMSCs could inhibit prostate inflammation in CBP rats, and the combination of LIPUS and microbubbles could promote the homing of BMSCs and improve the efficacy of BMSCs in CBP rats [88].

The above studies showed that the combination of LIPUS with microbubbles could promote the proliferation of BMSCs and promote the chondrogenic and osteogenic differentiation of BMSCs by up-regulating chondrogenic and osteogenic genes. In addition, the combination of LIPUS with microbubbles could promote the homing of BMSCs through the SDF-1/CXCR4 signaling pathway and enhance the therapeutic effects of BMSCs in myocardial infarction, AKI, and CBP. Although these studies demonstrate the effects of UTMD in BMSCs, the effects of UTMD in other sources of MSCs have not been reported. In addition, the above studies did not directly prove the cavitation effect of UTMD, and the mechanism of its effect on MSCs has not yet been elucidated. Further detailed studies need to be conducted to examine whether this cavitation effect will have a negative impact on MSCs.

## Conclusion and perspectives

In summary, the role of LIPUS in MSCs has been verified in many studies. LIPUS has been proved to promote the viability, proliferation, differentiation, and migration of MSCs. The combination of LIPUS and MSCs can be used for the treatment of various diseases. A new technique that enhances the efficacy of MSCs has also increasingly received attention from researchers. However, studies on the combination of LIPUS and MSCs are still in the preliminary stage; most of these are early cell experiments and a few are animal experiments. More animal experiments and clinical trials are needed in the future to prove the effectiveness of the use of LIPUS in MSC therapy. At the same time, the parameters of LIPUS reported in various studies are different, and different parameters have different effects on MSCs obtained from different sources. The exploration of more standard and suitable LIPUS parameters is one of the important objectives of future research. In addition, although MSCs have low immunogenicity, we need to study whether LIPUS would affect the immune rejection occurring after MSC transplantation, and whether it would cause MSCs to become tumorigenic; these safety issues should be focused upon in future studies. Finally, the mechanism of action of LIPUS and its combination with microbubbles during MSC transplantation is not completely clear, and more in-depth research is needed to examine these aspects in the future. It is believed that a greater amount of evidence would support the application of LIPUS in MSC therapy in the near future. LIPUS is expected to become an important auxiliary tool for the improvement in the efficacy of MSC therapy.

## Declarations

### Literature selection methods

The electronic databases of PubMed were searched through the combination of a series of logic keywords and text words including “ultrasound,” “low-intensity pulsed ultrasound,” “LIPUS,” “mesenchymal stem cells,” “mesenchymal stromal cells,” “MSCs,” and “stem cells.” The most recent electronic search was conducted in February 2022. The reference lists of retrieved articles and reviews were identified. Two researchers (PX and YS) reviewed all the retrieved abstracts and full texts independently. The eligibility criteria for this review were as follows: (a) the intervention comprised of LIPUS; (b) the articles discussed the application of LIPUS in MSCs; and (c) animal or human studies, and in vivo or in vitro studies. The exclusion criteria consisted of the following: (a) The duplicate and similar studies; (b) case reports and the conference abstract; (c) articles not with the MSCs, but the other type of stem cells; (d) articles with focused ultrasound.

## Data Availability

All data generated and/or analyzed during this study are included in this published article.

## Abbreviations

MSCs: Mesenchymal stem cells
LIPUS: Low-intensity pulsed ultrasound
OA: Osteoarthritis
FDA: Food and Drug Administration
NICE: National Institute for Health and Care Excellence
ACL: Anterior cruciate ligament
ERK1/2: Extracellular regulated protein kinases ½
PI3K: Phosphatidylinositide 3-kinases
GAG: Glycosaminoglycan
SDF-1: Stromal cell-derived factor-1
BMSCs: Bone marrow MSCs
ADSCs: Adipose-derived stem cells
AD-MSCs: Amnion-derived MSCs
DSCs: Dental stem cells
COL2: Type II collagen
TGF-β: Transforming growth factor-β
SOX9: Sex-determining region of Y chromosome-related high-mobility-group box gene 9
COL1: Type I collagen
mTOR: Mammalian target of rapamycin
ALP: Alkaline phosphatase
OCN: Osteocalcin
BMP-2: Bone morphogenetic protein-2
OPN: Osteopontin
PPAR-γ2: Proliferators-activated receptors-γ2
FABP4: Fatty acid-binding protein 4
MAPKK1 or MEK1: Mitogen-activated protein kinase kinase 1
ROCK: Rho-associated kinase
HGF: Hepatocyte growth factor
AFP: α-Fetoprotein
CK18: Cytokeratin 18; ALB: albumin
CXCR4: CXC chemokine receptor type 4
FAK: Focal adhesion kinase
BDNF: Brain-derived neurotrophic factor
NGF: Nerve growth factor
BSP: Bone sialoprotein
APN: Adiponectin
VE-cadherin: Vascular endothelial cadherin
HUVECs: Human umbilical vein endothelial cells
EGFR3: Epidermal growth factor receptor 3
NRG1: Neuregulin 1
EGR2: Early growth response protein 2
MBP: Myelin basic protein
PDLSCs: Periodontal ligament stem cells
DPSCs: Dental pulp stem cells
GMSCs: Gingival MSCs
SHEDs: Stem cell populations from human exfoliated deciduous teeth
DFPCs: Follicular cell progenitor cells
ABMSCs: Alveolar bone MSCs
SCAPs: Dental papilla stem cells
TGPCs: Tooth germ cells
NCT: Nucleostemin
LPS-PG: Porphyromonas gingivalis-derived LPS
IL-1β: Interleukin-1β
TWIST1: Twist family bHLH transcription factor-1
JNK: C-Jun N-terminal kinase
POI: Primary ovarian insufficiency
UTMD: Ultrasound-targeted microbubble destruction
AKI: Acute kidney injury
CBP: Chronic bacterial prostatitis
TNF-α: Tumor necrosis factor-α

## References

[1] Nurkovic J, Dolicanin Z, Mustafic F, Mujanovic R, Memic M, Grbovic V (2016). Mesenchymal stem cells in regenerative rehabilitation. J Phys Ther Sci.

[2] Lou S, Duan Y, Nie H, Cui X, Du J, Yao Y (2021). Mesenchymal stem cells: biological characteristics and application in disease therapy. Biochimie.

[3] Hosseini S, Taghiyar L, Safari F, Baghaban EM (2018). Regenerative medicine applications of mesenchymal stem cells.

[4] Poolman RW, Agoritsas T, Siemieniuk RA, Harris IA, Schipper IB, Mollon B (2017). Low intensity pulsed ultrasound (LIPUS) for bone healing: a clinical practice guideline. BMJ.

[5] Uddin S, Komatsu DE (2020). Therapeutic potential low-intensity pulsed ultrasound for osteoarthritis: pre-clinical and clinical perspectives. Ultrasound Med Biol.

[6] Lai WC, Iglesias BC, Mark BJ, Wang D (2021). Low-intensity pulsed ultrasound augments tendon, ligament, and bone-soft tissue healing in preclinical animal models: a systematic review. Arthroscopy.

[7] Tan Y, Guo Y, Reed-Maldonado AB, Li Z, Lin G, Xia SJ (2021). Low-intensity pulsed ultrasound stimulates proliferation of stem/progenitor cells: what we need to know to translate basic science research into clinical applications. Asian J Androl.

[8] Berebichez-Fridman R, Montero-Olvera PR (2018). Sources and clinical applications of mesenchymal stem cells: state-of-the-art review. Sultan Qaboos Univ Med J.

[9] Zhang B, Yin Y, Lai RC, Tan SS, Choo AB, Lim SK (2014). Mesenchymal stem cells secrete immunologically active exosomes. Stem Cells Dev.

[10] Wang Y, Chen X, Cao W, Shi Y (2014). Plasticity of mesenchymal stem cells in immunomodulation: pathological and therapeutic implications. Nat Immunol.

[11] Nitzsche F, Muller C, Lukomska B, Jolkkonen J, Deten A, Boltze J (2017). Concise review: MSC adhesion cascade-insights into homing and transendothelial migration. Stem Cells.

[12] Katsha AM, Ohkouchi S, Xin H, Kanehira M, Sun R, Nukiwa T (2011). Paracrine factors of multipotent stromal cells ameliorate lung injury in an elastase-induced emphysema model. Mol Ther.

[13] Padilla F, Puts R, Vico L, Raum K (2014). Stimulation of bone repair with ultrasound: a review of the possible mechanic effects. Ultrasonics.

[14] Zhang N, Chow SK, Leung KS, Cheung WH (2017). Ultrasound as a stimulus for musculoskeletal disorders. J Orthop Translat.

[15] Jiang X, Savchenko O, Li Y, Qi S, Yang T, Zhang W (2019). A review of low-intensity pulsed ultrasound for therapeutic applications. Ieee T Bio-Med Eng.

[16] Harrison A, Lin S, Pounder N, Mikuni-Takagaki Y (2016). Mode & mechanism of low intensity pulsed ultrasound (LIPUS) in fracture repair. Ultrasonics.

[17] Xia P, Shen S, Lin Q, Cheng K, Ren S, Gao M (2015). Low-intensity pulsed ultrasound treatment at an early osteoarthritis stage protects rabbit cartilage from damage via the integrin/focal adhesion kinase/mitogen-activated protein kinase signaling pathway. J Ultras Med.

[18] Xia P, Ren S, Lin Q, Cheng K, Shen S, Gao M (2015). Low-intensity pulsed ultrasound affects chondrocyte extracellular matrix production via an integrin-mediated p38 MAPK signaling pathway. Ultrasound Med Biol.

[19] Cheng K, Xia P, Lin Q, Shen S, Gao M, Ren S (2014). Effects of low-intensity pulsed ultrasound on integrin-FAK-PI3K/Akt mechanochemical transduction in rabbit osteoarthritis chondrocytes. Ultrasound Med Biol.

[20] Heckman JD, Ryaby JP, McCabe J, Frey JJ, Kilcoyne RF (1994). Acceleration of tibial fracture-healing by non-invasive, low-intensity pulsed ultrasound. J Bone Joint Surg Am.

[21] Kristiansen TK, Ryaby JP, McCabe J, Frey JJ, Roe LR (1997). Accelerated healing of distal radial fractures with the use of specific, low-intensity ultrasound. A multicenter, prospective, randomized, double-blind, placebo-controlled study. J Bone Joint Surg Am.

[22] Nolte PA, van der Krans A, Patka P, Janssen IM, Ryaby JP, Albers GH (2001). Low-intensity pulsed ultrasound in the treatment of nonunions. J Trauma.

[23] Food and Drug Administration. Approval order. 2000. www.fda.gov/ohrms/dockets/dailys/00/mar00/031300/aav0001.pdf.

[24] National Institute for Health and Clinical Excellence. Low-intensity pulsed ultrasound to promote fracture healing (interventional procedure guidance 374). 2010. www.nice.org.uk/guidance/ipg374.

[25] Palanisamy P, Alam M, Li S, Chow S, Zheng YP (2022). Low-intensity pulsed ultrasound stimulation for bone fractures healing: a review. J Ultrasound Med.

[26] Loyola-Sánchez A, Richardson J, Beattie KA, Otero-Fuentes C, Adachi JD, MacIntyre NJ (2012). Effect of low-intensity pulsed ultrasound on the cartilage repair in people with mild to moderate knee osteoarthritis: a double-blinded, randomized, placebo-controlled pilot study. Arch Phys Med Rehabil.

[27] Jia L, Wang Y, Chen J, Chen W (2016). Efficacy of focused low-intensity pulsed ultrasound therapy for the management of knee osteoarthritis: a randomized, double blind, placebo-controlled trial. Sci Rep.

[28] Ying ZM, Lin T, Yan SG (2012). Low-intensity pulsed ultrasound therapy: a potential strategy to stimulate tendon-bone junction healing. J Zhejiang Univ Sci B.

[29] Xie S, Jiang X, Wang R, Xie S, Hua Y, Zhou S (2019). Low-intensity pulsed ultrasound promotes the proliferation of human bone mesenchymal stem cells by activating PI3K/AKt signaling pathways. J Cell Biochem.

[30] Amini A, Chien S, Bayat M (2020). Impact of ultrasound therapy on stem cell differentiation—a systematic review. Curr Stem Cell Res T.

[31] Cui JH, Park K, Park SR, Min BH (2006). Effects of low-intensity ultrasound on chondrogenic differentiation of mesenchymal stem cells embedded in polyglycolic acid: an in vivo study. Tissue Eng.

[32] Wei FY, Leung KS, Li G, Qin J, Chow SK, Huang S (2014). Low intensity pulsed ultrasound enhanced mesenchymal stem cell recruitment through stromal derived factor-1 signaling in fracture healing. PLoS ONE.

[33] Lim K, Kim J, Seonwoo H, Park SH, Choung PH, Chung JH (2013). In vitro effects of low-intensity pulsed ultrasound stimulation on the osteogenic differentiation of human alveolar bone-derived mesenchymal stem cells for tooth tissue engineering. Biomed Res Int.

[34] Lee HJ, Choi BH, Min BH, Park SR (2007). Low-intensity ultrasound inhibits apoptosis and enhances viability of human mesenchymal stem cells in three-dimensional alginate culture during chondrogenic differentiation. Tissue Eng.

[35] Ling L, Wei T, He L, Wang Y, Wang Y, Feng X (2017). Low-intensity pulsed ultrasound activates ERK1/2 and PI3K-Akt signalling pathways and promotes the proliferation of human amnion-derived mesenchymal stem cells. Cell Prolif.

[36] Xia P, Wang X, Wang Q, Wang X, Lin Q, Cheng K (2021). Low-intensity pulsed ultrasound promotes autophagy-mediated migration of mesenchymal stem cells and cartilage repair. Cell Transplant.

[37] Charbord P (2010). Bone marrow mesenchymal stem cells: historical overview and concepts. Hum Gene Ther.

[38] Yang X, Wu Y, Li J, Yin W, An Y, Wang Y (2019). A pilot study of parameter-optimized low-intensity pulsed ultrasound stimulation for the bone marrow mesenchymal stem cells viability improvement. Comput Math Methods Med.

[39] Aliabouzar M, Lee SJ, Zhou X, Zhang GL, Sarkar K (2018). Effects of scaffold microstructure and low intensity pulsed ultrasound on chondrogenic differentiation of human mesenchymal stem cells. Biotechnol Bioeng.

[40] Zhi Z, Na T, Jue W, Zhihe Z (2016). Lijun T [Effects of pulsed ultrasound and pulsed electromagnetic field on the extracellular matrix secretion of rat bone marrow mesenchymal stem cell pellets in chondrogenesis]. Hua Xi Kou Qiang Yi Xue Za Zhi.

[41] Xia P, Wang X, Qu Y, Lin Q, Cheng K, Gao M (2017). TGF-β1-induced chondrogenesis of bone marrow mesenchymal stem cells is promoted by low-intensity pulsed ultrasound through the integrin-mTOR signaling pathway. Stem Cell Res Ther.

[42] Wang X, Lin Q, Zhang T, Wang X, Cheng K, Gao M (2019). Low-intensity pulsed ultrasound promotes chondrogenesis of mesenchymal stem cells via regulation of autophagy. Stem Cell Res Ther.

[43] An Y, Song Y, Wang Z, Wang J, Wu G, Zhu G (2018). Effect of low-intensity pulsed ultrasound on the biological behaviors of bone marrow mesenchymal stem cells on titanium with different surface topographies. Am J Transl Res.

[44] Kusuyama J, Bandow K, Shamoto M, Kakimoto K, Ohnishi T, Matsuguchi T (2014). Low intensity pulsed ultrasound (LIPUS) influences the multilineage differentiation of mesenchymal stem and progenitor cell lines through ROCK-Cot/Tpl2-MEK-ERK signaling pathway. J Biol Chem.

[45] Li F, Liu Y, Cai Y, Li X, Bai M, Sun T (2018). Ultrasound irradiation combined with hepatocyte growth factor accelerate the hepatic differentiation of human bone marrow mesenchymal stem cells. Ultrasound Med Biol.

[46] Chen J, Jiang J, Wang W, Qin J, Chen J, Chen W (2019). Low intensity pulsed ultrasound promotes the migration of bone marrow- derived mesenchymal stem cells via activating FAK-ERK1/2 signalling pathway. Artif Cells Nanomed Biotechnol.

[47] Ning GZ, Song WY, Xu H, Zhu RS, Wu QL, Wu Y (2019). Bone marrow mesenchymal stem cells stimulated with low-intensity pulsed ultrasound: better choice of transplantation treatment for spinal cord injury: treatment for SCI by LIPUS-BMSCs transplantation. Cns Neurosci Ther.

[48] Wang Y, Li J, Zhou J, Qiu Y, Song J (2022). Low-intensity pulsed ultrasound enhances bone marrow-derived stem cells-based periodontal regenerative therapies. Ultrasonics.

[49] Xia P, Wang X, Wang Q, Wang X, Lin Q, Cheng K (2021). Low-intensity pulsed ultrasound promotes autophagy-mediated migration of mesenchymal stem cells and cartilage repair. Cell Transplant.

[50] Bacakova L, Zarubova J, Travnickova M, Musilkova J, Pajorova J, Slepicka P (2018). Stem cells: their source, potency and use in regenerative therapies with focus on adipose-derived stem cells—a review. Biotechnol Adv.

[51] Wang Y, Jiang L, Xu T, Su Z, Guo X, Tu J (2019). p38 MAPK signaling is a key mediator for low-intensity pulsed ultrasound (LIPUS) in cultured human omental adipose-derived mesenchymal stem cells. Am J Transl Res.

[52] Huang D, Gao Y, Wang S, Zhang W, Cao H, Zheng L (2020). Impact of low-intensity pulsed ultrasound on transcription and metabolite compositions in proliferation and functionalization of human adipose-derived mesenchymal stromal cells. Sci Rep.

[53] Jiang T, Xu T, Gu F, Chen A, Xiao Z, Zhang D (2012). Osteogenic effect of low intensity pulsed ultrasound on rat adipose-derived stem cells in vitro. J Huazhong Univ Sci Technol Med Sci.

[54] Yue Y, Yang X, Wei X, Chen J, Fu N, Fu Y (2013). Osteogenic differentiation of adipose-derived stem cells prompted by low-intensity pulsed ultrasound. Cell Prolif.

[55] Fu N, Yang X, Ba K, Fu Y, Wei X, Yue Y (2013). Low-intensity pulsed ultrasound induced enhanced adipogenesis of adipose-derived stem cells. Cell Prolif.

[56] Kang PL, Huang HH, Chen T, Ju KC, Kuo SM (2019). Angiogenesis-promoting effect of LIPUS on hADSCs and HUVECs cultured on collagen/hyaluronan scaffolds. Mater Sci Eng C Mater Biol Appl.

[57] Yue Y, Yang X, Zhang L, Xiao X, Nabar NR, Lin Y (2016). Low-intensity pulsed ultrasound upregulates pro-myelination indicators of Schwann cells enhanced by co-culture with adipose-derived stem cells. Cell Prolif.

[58] Chen C, Zhang T, Liu F, Qu J, Chen Y, Fan S (2019). Effect of low-intensity pulsed ultrasound after autologous adipose-derived stromal cell transplantation for bone-tendon healing in a rabbit model. Am J Sports Med.

[59] Nasb M, Liangjiang H, Gong C, Hong C (2020). Human adipose-derived Mesenchymal stem cells, low-intensity pulsed ultrasound, or their combination for the treatment of knee osteoarthritis: study protocol for a first-in-man randomized controlled trial. BMC Musculoskelet Disord.

[60] Sharpe PT (2016). Dental mesenchymal stem cells. Development.

[61] Gan L, Liu Y, Cui D, Pan Y, Zheng L, Wan M (2020). Dental tissue-derived human mesenchymal stem cells and their potential in therapeutic application. Stem Cells Int.

[62] El-Bialy T, Alhadlaq A, Lam B (2012). Effect of therapeutic ultrasound on human periodontal ligament cells for dental and periodontal tissue engineering. Open Dent J.

[63] Hu B, Zhang Y, Zhou J, Li J, Deng F, Wang Z (2014). Low-intensity pulsed ultrasound stimulation facilitates osteogenic differentiation of human periodontal ligament cells. PLoS ONE.

[64] Kusuyama J, Nakamura T, Ohnishi T, Eiraku N, Noguchi K, Matsuguchi T (2017). Low-intensity pulsed ultrasound (LIPUS) promotes BMP9-induced osteogenesis and suppresses inflammatory responses in human periodontal ligament-derived stem cells. J Orthop Trauma.

[65] Wang Y, Li J, Qiu Y, Hu B, Chen J, Fu T (2018). Low-intensity pulsed ultrasound promotes periodontal ligament stem cell migration through TWIST1-mediated SDF-1 expression. Int J Mol Med.

[66] Gao Q, Walmsley AD, Cooper PR, Scheven BA (2016). Ultrasound stimulation of different dental stem cell populations: role of mitogen-activated protein kinase signaling. J Endodont.

[67] Gao Q, Cooper PR, Walmsley AD, Scheven BA (2017). Role of Piezo channels in ultrasound-stimulated dental stem cells. J Endodont.

[68] El-Bialy T, Alhadlaq A, Wong B, Kucharski C (2014). Ultrasound effect on neural differentiation of gingival stem/progenitor cells. Ann Biomed Eng.

[69] Liu QW, Huang QM, Wu HY, Zuo GS, Gu HC, Deng KY (2021). Characteristics and therapeutic potential of human amnion-derived stem cells. Int J Mol Sci.

[70] Ling L, Feng X, Wei T, Wang Y, Wang Y, Zhang W (2017). Effects of low-intensity pulsed ultrasound (LIPUS)-pretreated human amnion-derived mesenchymal stem cell (hAD-MSC) transplantation on primary ovarian insufficiency in rats. Stem Cell Res Ther..

[71] Lu LL, Liu YJ, Yang SG, Zhao QJ, Wang X, Gong W (2006). Isolation and characterization of human umbilical cord mesenchymal stem cells with hematopoiesis-supportive function and other potentials. Haematologica.

[72] Batsali AK, Kastrinaki MC, Papadaki HA, Pontikoglou C (2013). Mesenchymal stem cells derived from Wharton's Jelly of the umbilical cord: biological properties and emerging clinical applications. Curr Stem Cell Res Ther.

[73] Cho PS, Messina DJ, Hirsh EL, Chi N, Goldman SN, Lo DP (2008). Immunogenicity of umbilical cord tissue derived cells. Blood.

[74] Li T, Xia M, Gao Y, Chen Y, Xu Y (2015). Human umbilical cord mesenchymal stem cells: an overview of their potential in cell-based therapy. Expert Opin Biol Th.

[75] Yoon JH, Roh EY, Shin S, Jung NH, Song EY, Lee DS (2009). Introducing pulsed low-intensity ultrasound to culturing human umbilical cord-derived mesenchymal stem cells. Biotechnol Lett.

[76] Chen X, Jin X, Huang D, Xing S, Wang H, Zhuo Z (2019). Effects of low-intensity pulsed ultrasound on proliferation and chondrogenic differentiation of human umbilical cord-derived mesenchymal stem celIs. Zhong Guo Kang Fu Yi Xue Za Zhi.

[77] Bekeredjian R, Katus H, Kuecherer H (2006). Therapeutic use of ultrasound targeted microbubble destruction: a review of non-cardiac applications. Ultraschall in der Medizin - European Journal of Ultrasound.

[78] Cui H, Zhu Q, Xie Q, Liu Z, Gao Y, He Y (2020). Low intensity ultrasound targeted microbubble destruction assists MSCs delivery and improves neural function in brain ischaemic rats. J Drug Target.

[79] Qian J, Wang L, Li Q, Sha D, Wang J, Zhang J (2018). Ultrasound-targeted microbubble enhances migration and therapeutic efficacy of marrow mesenchymal stem cell on rat middle cerebral artery occlusion stroke model. J Cell Biochem.

[80] Song X, Zhu H, Jin L, Wang J, Yang Q, Jin P (2009). Ultrasound-mediated microbubble destruction enhances the efficacy of bone marrow mesenchymal stem cell transplantation and cardiac function. Clin Exp Pharmacol Physiol.

[81] Sun T, Gao F, Li X, Cai Y, Bai M, Li F (2018). A combination of ultrasound-targeted microbubble destruction with transplantation of bone marrow mesenchymal stem cells promotes recovery of acute liver injury. Stem Cell Res Ther.

[82] Sun Z, Xie Y, Lee RJ, Chen Y, Jin Q, Lv Q (2020). Myocardium-targeted transplantation of PHD2 shRNA-modified bone mesenchymal stem cells through ultrasound-targeted microbubble destruction protects the heart from acute myocardial infarction. Theranostics.

[83] Wei X, Zheng Y, Zhang W, Tan J, Zheng H (2021). Ultrasound-targeted microbubble destruction-mediated Galectin-7-siRNA promotes the homing of bone marrow mesenchymal stem cells to alleviate acute myocardial infarction in rats. Int J Mol Med.

[84] Aliabouzar M, Zhang LG, Sarkar K (2016). Lipid Coated Microbubbles and Low Intensity Pulsed Ultrasound Enhance Chondrogenesis of Human Mesenchymal Stem Cells in 3D Printed Scaffolds. Sci Rep.

[85] Osborn J, Aliabouzar M, Zhou X, Rao R, Zhang LG, Sarkar K (2019). Enhanced osteogenic differentiation of human mesenchymal stem cells using microbubbles and low intensity pulsed ultrasound on 3D printed scaffolds. Adv Biosyst.

[86] Li L, Wu S, Liu Z, Zhuo Z, Tan K, Xia H (2015). Ultrasound-targeted microbubble destruction improves the migration and homing of mesenchymal stem cells after myocardial infarction by upregulating SDF-1/CXCR4: a pilot study. Stem Cells Int.

[87] Wang G, Zhang Q, Zhuo Z, Wu S, Xu Y, Zou L (2016). Enhanced homing of CXCR-4 modified bone marrow-derived mesenchymal stem cells to acute kidney injury tissues by micro-bubble–mediated ultrasound exposure. Ultrasound Med Biol.

[88] Yi S, Han G, Shang Y, Liu C, Cui D, Yu S (2016). Microbubble-mediated ultrasound promotes accumulation of bone marrow mesenchymal stem cell to the prostate for treating chronic bacterial prostatitis in rats. Sci Rep.

